# Sustained, Multifaceted Improvements in Mental Well-Being Following Psychedelic Experiences in a Prospective Opportunity Sample

**DOI:** 10.3389/fpsyt.2021.647909

**Published:** 2021-06-29

**Authors:** Keri Mans, Hannes Kettner, David Erritzoe, Eline C. H. M. Haijen, Mendel Kaelen, Robin L. Carhart-Harris

**Affiliations:** Centre for Psychedelic Research, Division of Psychiatry, Department of Brain Sciences, Imperial College London, London, United Kingdom

**Keywords:** mental well-being, psychedelics, mental health, naturalistic setting, survey study, longitudinal, exploratory factor analysis, early intervention

## Abstract

In the last 15 years, psychedelic substances, such as LSD and psilocybin, have regained legitimacy in clinical research. In the general population as well as across various psychiatric populations, mental well-being has been found to significantly improve after a psychedelic experience. Mental well-being has large socioeconomic relevance, but it is a complex, multifaceted construct. In this naturalistic observational study, a comprehensive approach was taken to assessing well-being before and after a taking a psychedelic compound to induce a “psychedelic experience.” Fourteen measures of well-being related constructs were included in order to examine the breadth and specificity of change in well-being. This change was then analysed to examine clusters of measures changing together. Survey data was collected from volunteers that intended to take a psychedelic. Four key time points were analysed: 1 week before and 2 weeks, 4 weeks, and 2 years after the experience (*N* = 654, *N* = 315, *N* = 212, and *N* = 64, respectively). Change on the included measures was found to cluster into three factors which we labelled: 1) “Being well”, 2) “Staying well,” and 3) “Spirituality.” Repeated Measures Multivariate Analysis of Variance revealed all but the spirituality factor to be improved in the weeks following the psychedelic experience. Additional Mixed model analyses revealed selective increases in *Being Well* and *Staying Well* (but not *Spirituality*) that remained statistically significant up to 2 years post-experience, albeit with high attrition rates. *Post-hoc* examination suggested that attrition was not due to differential acute experiences or mental-health changes in those who dropped out vs. those who did not. These findings suggest that psychedelics can have a broad, robust and sustained positive impact on mental well-being in those that have a prior intention to use a psychedelic compound. Public policy implications are discussed.

## Introduction

Mental well-being^1^ is a broad construct that includes both positive mood and good general functioning (1). There is a reliable inverse relationship between mental well-being and mental illness (2) and evidence suggests that this relationship is continuous rather than discrete (3, 4). Mental health problems are currently among the leading causes of disability worldwide, with substantial personal, social, and economic costs attached (4). Efforts to promote and maintain well-being should therefore be considered a priority area for policy makers and healthcare systems (4, 5), and indeed well-being has received increasing interest over the past decades, with efforts to recognise, improve and protect it (2, 4, 6–10). The limitations of the default psychiatric strategy of reactively intervening post-diagnosis are increasingly recognised, this approach being unlikely to provide a solution to current and future individual and population-level mental health problems (11). Consistent with this view, the World Health Organization (WHO) highlights the need for a comprehensive perspective on mental health and implementation of proactive and preventative strategies (10).

Various interventions aiming to promote and protect mental health are currently available, ranging from pharmacotherapy and various psychotherapies, to mindfulness and life skills training (6, 12–14). It is suggested that, besides alleviating symptoms in clinical populations, initiatives and interventions for people that are already “well” could serve to further promote wellness and mitigate risk of mental illness (13). However, current interventions have various limitations and thus new safe, affordable and effective ones are needed (15). One such novel intervention is psychedelic (“mind-manifesting”) therapy, i.e., supervised psychedelic drug experiences bookended by psychological support (16–19). Classic psychedelic drugs^2^ such as LSD, DMT (dimethyltryptamine), and psilocybin (4-phosphoryloxy-N,N-dimethyltryptamine), are (non-selective) serotonin 2A receptor (5-HT2AR) agonist drugs with potent perception and consciousness-altering properties (20, 21).

Recent studies in clinical and healthy populations have revealed marked, rapid, and lasting (therapeutic) effects from just one/two psychedelic dosing sessions, which include improvements in well-being (22). For example, a single dose of psilocybin received by healthy psychedelic-naïve participants was associated with increased ratings of well-being and life satisfaction 14 months later (23). Reduced rates of psychological distress and suicidality have been observed in large cross-sectional population studies (24, 25), and increases in optimism, trait openness, mood, psychological flexibility, mindfulness capacities, and subjective well-being have been found in both controlled and naturalistic prospective studies (23, 26–39). These findings are suggestive of the prophylactic value of psychedelic therapy (40) as well as its relevance for positive psychology (41)—while being mindful of the context dependency of these outcomes—where, for example, preparedness and psychological support are thought to be essential (42). Despite the accumulating evidence for therapeutic and generalised mental health enhancing potential of psychedelic compounds, including in populations not (currently) suffering from psychopathology, it remains unclear which components of well-being are most sensitive to change after exposure to psychedelics. Hence, a deeper examination of the nature of this relationship is needed (43), which may also guide choices of scales to use in future studies.

Well-being is a complex, multi-faceted construct. Examining well-being encompasses a complexity that is two-fold (44, 45). First is the difficulty in narrowing the broad construct of well-being down to a unified, generally accepted definition—which has been a topic of concern for academics and philosophers from Aristotle to the present day [(1, 44); also see Supplementary Table 1]. The most widely accepted approach currently defines well-being as a multidimensional construct comprising both feeling good or “*hedonia*” from Diener's (46) model and functioning well (1, 47) or “*eudaimonia*” from various models [e.g., (48–50)]. Although there is substantial overlap between these models, contemporary researchers have not reached consensus on what exactly constitutes eudaimonia (51). Recently, scholars in the field of positive psychology have endeavoured to approach the full construct of well-being, operationally defining it as “positive mental health” (52) or “flourishing” (3), yet no single definition has been generally accepted yet.

The second aspect that reflects its complexity, is the divergence of (self-report) measures designed to assess well-being [e.g., (45, 53–55)]. This creates ambiguity, bias, and inconsistency in assessment and confuses the development of effective (preventive) interventions (45). Varying subdivisions, if included in the measures, may either place the construct on a continuum between depression and happiness, or rather divide it into subordinate constructs such as positive affect, self-acceptance, personal growth, interpersonal relationships, and purpose in life (55). Furthermore, mental health assessment has a clinical heritage, and thus many of the available measures have been influenced by clinical classification systems (e.g., DSM or ICD) that may not necessarily translate well to the general population. An increasingly accepted position is to view well-being as more than merely the absence of psychopathology, just as health is more than the absence of disease [i.e., (1, 56, 57)]. To provide perspective on a person's general mental health, rather than merely identifying disorders, assessment tools should therefore comprehensively approach well-being in line with this view. Is it suggested that this may be best approached by combining measures of underlying and related constructs (3, 52, 58). This comprehensive approach, with well-being as an umbrella term, is the approach followed in the current study.

Which aspects of well-being are most relevant to psychedelics? Spirituality and purpose in life are debated components of well-being (55), but are often cited in relation to psychedelics (31, 59). A recent study that assessed psilocybin experiences in healthy individuals, found positive enduring changes on measures of gratitude, life meaning/purpose, coping, and interpersonal closeness, particularly when the psychedelic experience was combined with meditation and spiritual practises (60). Further, the construct of connectedness, i.e., a sense of feeling connected to oneself, others, and the world, has also been linked to the long-term positive psychological effects of psychedelics (61). Taken together, there are good reasons to include factors such as connectedness, meaning or purpose in life, and spirituality, when assessing the broad impact of psychedelics on well-being.

The present study endeavoured to comprehensively assess the effects of psychedelics on well-being by conducting a naturalistic, observational study using opportunity sampling and online data collection. Treating well-being as an umbrella construct, we included various measures pertaining to it. We then factorised these into a smaller number of covarying components and examined which of them, and the specific measures that load onto them, were most sensitive to change via a psychedelic. One motivation for doing this was to inform future decisions about which measures to include in future psychedelic studies, given the importance of parsimony and efficiency in study design. Our main hypothesis was that psychedelics would have a comprehensive impact on well-being and secondary, exploratory analyses assessed differential sensitivity to change. Study outcomes from this opportunity sample may have implications for the potential prophylactic value of psychedelics via their capacity to promote and maintain (facets of) mental well-being.

## Methods

### Design

The current study used data from an anonymous, prospective cohort study that was conducted between March and November 2017 through the online platform “*Psychedelic Survey*” (www.psychedelicsurvey.com). Survey data on individuals' experiences before and after using a psychedelic compound (i.e., undergoing the psychedelic experience) and personal characteristics were collected. The opportunity sampling and web-based data collection provided an opportunity for collecting a large amount of data in a non-controlled, naturalistic, and observational manner. For an overview of the main findings and methods, see Haijen et al. (32). Only measures that are of relevance to the research questions of the current study will be described below. Follow-up data, collected between July and August 2019, was used to assess longer term effects. Imperial College Research Ethics Committee (ICREC) gave a favourable opinion and the Joint Research Compliance Office (JRCO) at Imperial College London approved the study.

### Participants

The sample consisted of individuals who planned to undergo a psychedelic experience through their own initiative. All participants gave informed consent prior to their participation. This was done electronically, by ticking a box to declare that they had read and understood the consent form information. The population was equal to the 2018 study (32).

Inclusion criteria were: >18 years old, a good comprehension of the English language, and the intention to take one of the following psychedelic drugs in the near future: psilocybin/magic mushrooms/truffles, LSD/1P-LSD, ayahuasca, DMT/5-MeO-DMT, *salvia divinorum*, mescaline, or iboga/ibogaine. The response options for salvia divinorum and iboga/ibogaine were given, but no participants who indicated use of them at baseline were included in the study (see Supplementary Table 2). No encouragement of drug use was given whatsoever by the researchers or survey. The non-controlled manner also meant no interference or provision of information regarding the compounds used.

**Figure 1:**
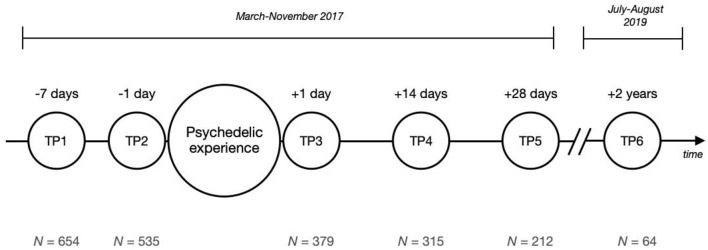
Survey study timeline. The small circles represent the six time points (TP) of measurement. Above each circle the time in reference to the psychedelic experience is shown; below each circle the corresponding sample size (*N*).

The final sample sizes were *N* = 654 (baseline), *N* = 535, *N* = 379, *N* = 315, *N* = 212, and *N* = 64 (final follow-up), respectively, for the six survey time points. See Figure 1 for all time points.

### Procedure

The study was advertised online using social media outlets, such as Facebook, Twitter, email newsletters, and online drug forums. Once participants signed up, they were included in an emailing system which sent out email reminders at specific times depending on the anticipated date of the psychedelic experience, as provided by the participants during the sign-up process. Emails contained links to the relevant surveys, which were implemented and hosted on the online service system “*Survey Gizmo*,” featuring security protection of responses. The study consisted of six surveys completed at different points in time, before and after the day the psychedelic compound was taken, as depicted in Figure 1.

### Measures

#### Type of Measures

Measures incorporated in the initial study (32) that are conceptually related to the broader construct of well-being and that were measured at baseline, as well as 2 weeks, 4 weeks, and 2 years after the experience, were included as dependent variables in the main analyses for the current study. These 14 measures were the following: the Warwick-Edinburgh Mental Well-being Scale (WEMWBS); Quick Inventory of Depressive Symptoms, 16-item self-report (QIDS-SR_16_); Rosenberg Self-Esteem Scale (RSE); Revised Life Orientation Test (LOT-R); Ten-item Personality Inventory, Emotional Stability subscale (TIPI-ES); Meaning in Life Questionnaire, Presence subscale (MLQ-P); Acceptance and Action Questionnaire II (AAQ-II); Brief Resilience Scale (BRS); Revised Cognitive and Affective Mindfulness Scale (CAMS-R); Social Connectedness Scale (SCS); Gratitude Questionnaire (GQ-6); Spiritual Transcendence Scale, Universality subscale (STS-U); Spiritual and Religious Attitudes in Dealing with Illness, modified short form, Trust subscale (SpREUK-SF-T); and the Santa Clara Brief Compassion Scale (SCBCS). The dependent variables were all continuous measures, sampled over four different time points; “time” was the sole independent variable. Three acute variables, measured 1 day after the psychedelic experience, were included for additional analyses. These included: the Challenging Experience Questionnaire (CEQ), the Mystical Experience Questionnaire (MEQ), and the Emotional Breakthrough Inventory (EBI).

#### Selection of Outcome Measures for Main Analyses

Previous literature guided the selection of 14 measures closely related to the multidimensional construct of well-being, including the dimensions of *hedonia* (positive affect and life satisfaction) and *eudaimonia* (psychological well-being, self-realisation, and interpersonal relationships). Table 1 offers an overview of these measures. The Warwick-Edinburgh Mental Well-being scale (WEMWBS) is a questionnaire designed to capture a generic conception of mental well-being capturing three key concepts: positive affect, psychological functioning, and interpersonal relationships, but summed as one factor representing a single underlying construct. It served as the primary outcome measure in this study. The 13 secondary measures were selected because of their conceptual closeness to well-being (Supplementary Table 1) as well as specific relevance to phenomena associated with psychedelic experiences such as spirituality, connectedness, meaning in life and compassion (61, 75). An extended version of Table 1, including more characteristics of the measures, can be found in Supplementary Table 3.

**Table 1:** Included outcome measures for the main analyses.

	**Measure**	**Construct measured [and subscale(s) used]^a^**
Primary measure	1. **WEMWBS** The Warwick-Edinburgh Mental Well-being scale (55)	**Mental well-being**
Secondary measures	1. **QIDS-SR**_**16**_ Quick Inventory of Depression Symptoms, 16-item Self Report (62)	**Depression symptoms** All subscales: sleep, sad, appetite, concentration, sleep, view of oneself, suicide, interest, energy, psychomotor
	2. **RSE** Rosenberg Self-Esteem Scale (63, 64)	**Self-esteem**
	3. **LOT-R** Revised Life Orientation Test (65)	**Optimism**
	4. **TIPI** Ten-item Personality Inventory (66)	**Personality** 1/5 subscales: Emotional stability (TIPI-ES)
	5. **MLQ** Meaning in Life Questionnaire (67)	**Meaning in life** 1/2 subscales: Presence (MLQ-P)
	6. **AAQ-II** Acceptance and Action Questionnaire-II (68)	**Psychological inflexibility and experiential avoidance**
	7. **BRS** Brief Resilience Scale (69)	**Resilience**
	8. **CAMS-R** Revised Cognitive and Affective Mindfulness Scale (70)	**Mindfulness**
	9. **SCS** Social Connectedness Scale (71)	**Social connectedness**
	10. **GQ-6** Gratitude Questionnaire (72)	**Gratitude**
	11. **STS** Spiritual Transcendence Scale (73)	**Spirituality** 1/3 subscales: Universality (STS-U)
	12. **SpREUK-SF** Spiritual and Religious Attitudes in Dealing with Illness—modified short form (74)	**Spirituality** 1/5 subscales: Trust (SpREUK-SF-T)
	13. **SCBCS** Santa Clara Brief Compassion Scale (75)	**Compassion**

a *All outcome measures are continuous (interval or ratio) measures*.

### Statistical Analyses

#### Planned Analyses

Correlation analyses, general linear models (GLMs), and dimension reduction analyses were carried out to investigate the broader construct of well-being in the context of psychedelic drug use. Statistical analyses were run in IBM SPSS 25, using a conventional alpha level of α = 0.05 and two-tailed hypothesis testing.

Data from survey time points 1, 4, and 5 (TP1, TP4, and TP5) was used in the main analyses; aimed at investigating facets underlying well-being that are most susceptible to change after a psychedelic experience and determining a factor structure of this change. Follow-up data from time point 6 (TP6) was used to explore longer-term implications of this model. Demographic information collected at baseline, including: age, gender, employment status, history of psychiatric illnesses, and (frequency of) previous drug use, was assessed in order to gain insight into the characteristics of the convenience sample. In additional analyses, data from survey time point 3 (TP3) was also used with the aim of assessing potential attrition bias.

#### Assumptions

Variables were tested on appropriate assumptions [e.g., linearity, (multivariate) normality, homogeneity, sphericity, and multicollinearity] before being entered into the analyses. Corrections were applied when sphericity could not be assumed.

#### Correlations

Correlation analyses were carried out to explore whether the selected secondary measures were indeed (closely) related to the primary measure, as well as to investigate covariance between the secondary measures. These analyses were carried out in an explorative manner, not aiming to serve hypothesis confirming purposes. Note that simple pairwise comparisons were therefore conducted, in which no corrections were made for multiple comparisons. Included measures were assumed to be highly interrelated, for which a conventionally used Bonferroni correction would arguably be too conservative (76, 77).

#### GLM

In order to assess psychedelic-induced changes on the assessed outcome measures, all 14 measures were included as dependent variables in a repeated-measures multivariate analysis of variance (RM MANOVA), to minimise errors associated with multiple comparisons, with time as within-subjects factor. Cases were excluded listwise, meaning that complete data was required for all three time points for a participant's scores to be included in these analyses.

For multivariate analyses, Pillai's trace was used, which is suggested to be the most robust test statistic for multivariate analysis of variance (78).

#### Dimension Reduction

Exploratory factor analysis (EFA) was carried out on standardised change scores between TP1 and TP5 to investigate factors or constructs underlying well-being that appeared to change together over time in relation to a psychedelic experience. Prior to running the EFA, several cheques were applied to ensure the suitability of data. First, adequacy of sample size was assessed using the Kaiser-Meyer-Olkin (KMO) test statistic [≥0.60; (79)] and Bartlett's test of sphericity achieving statistical significance at an alpha level of α = 0.001.

Furthermore, the multicollinearity determinant of the matrix had to be |R| >0.0001. Regarding residual correlations, the proportion of non-redundant residuals with absolute values >0.05 was deemed acceptable as long as it was below 50%. The proportion of shared variance within each variable (communalities) was not to exceed 0.70 (77).

The type of EFA used was principal axis factoring (PAF). As the constructs included in this factor analysis were assumed to correlate, the selected rotation form was oblique (Promax) rotation with a Kappa value of 4. The scree plot and eigenvalues, following Kaiser's criterion of >1, were used to determine the number of factors to extract (77).

Cases were excluded listwise, resulting in a sample size of *N* = 185 and a case to variable ratio of 1:12.33. Ratios of 1:10 and higher are considered sufficient (80).

Cronbach's alpha was computed as an index for reliability for each extracted factor.

#### Factor Scores

For each of the three time points, factor scores were computed: sum scores for all measures that were found to constitute a measure were added and divided by the number of scales constituting that factor. Factor scores were than normalised so they all ranged between 0 and 1. This allowed for comparison between factors and across the three time points.

Another RM MANOVA was run to assess changes of the factors over time, using pairwise *post-hoc* comparisons (within subject contrasts) Bonferroni adjusted for multiple comparisons.

#### Follow-Up Data Analysis

Due to the large attrition at the 2-year endpoint, a Linear Mixed Model was chosen instead of GLM Repeated Measures analyses in order to test changes across the entire duration of the study, leveraging its ability to better accommodate missing data. An “Unstructured” covariance structure was used, which resulted in the best model fit (lowest Akaike's Information Criterion). A random intercept was included.

#### Attrition Bias

For addressing potential attrition biases in this sample, differences in scores on five selected variables were assessed for individuals completing vs. not completing the final timepoint (TP6). Two of these variables included factor change scores from TP1 to TP5. The direction of the change from baseline to the 4-week endpoint on two of our well-being factors might inform on how positive/negative one rated their experience, which could, in turn, be an incentive to either or not drop out. The remaining three variables assessed were subjective measures: the Challenging Experience Questionnaire (CEQ), the Mystical Experience Questionnaire (MEQ), and the Emotional Breakthrough Inventory (EBI), again variables that may affect how positive one rates the experience, resulting in reason to either or not drop out. The extent of challenging experiences was expected to be an important indicator of potential biases, thus the CEQ subscales (Fear, Grief, Physical Distress, Insanity, Isolation, Death, and Paranoia) were also assessed in relation to well-being change scores.

#### Guidelines Followed

Interpretation of the effect sizes of Pearson correlation coefficients, or the strength of the relationship amongst continuous variables, was performed in accordance with the guidelines of *r* = 0.10, *r* = 0.30, and *r* = 0.50, respectively, representing a small, medium, and large effect. Guidelines used for the effect size of partial eta squared were: $\eta_{{\text{p}}^{2}}$ = 0.01 (small), $\eta_{{\text{p}}^{2}}$ = 0.09 (medium), and $\eta_{{\text{p}}^{2}}$ = 0.25 (large) (77, 81, 82). This effect size statistic is used for interpreting the strength of the proportion of total variance explained by a variable, which is not explained by other variables. It should be noted that these guidelines are merely followed as rules of thumb.

## Results

### Demographics

An overview of demographic information at baseline is provided in Table 2. See Haijen et al. (32) for further demographics.

**Table 2:** Demographic data collected at baseline (time point 1; TP1).

**Demographic**		***M* (*SD*)**	***N***	**% of baseline frequency**
Age and gender	All	28.9 (10.45)	654	100%
	Male	28.0 (10.1)	485	74.2%
	Female	31.56 (11.2)	165	25.2%
	Other	23.0 (2.8)	4	0.6%
Employment status	Student		256	39.1%
	Unemployed		53	8.1%
	Part-time job		98	15.0%
	Full-time job		237	36.2%
	Retired		10	1.5%
Nationality	United States		199	30.4%
	United Kingdom		128	19.6%
	Denmark		60	9.2%
	Germany		32	4.9%
	Canada		32	4.9%
	The Netherlands		15	2.3%
	Other (49 other)		188	28.7%
Psychiatric history	Ever been diagnosed with at least one psychiatric illness^a^		214	32.7%
	Never been diagnosed with a psychiatric illness^a^		440	67.3%
Previous drug use	Psychedelics^b^–used at least once before		592	90.5%
	Psychedelics^b^–never used		62	9.5%
	Other drugs^c^–used at least once before		620	94.8%
	Other drugs^c^–never used		34	5.2%

a *Including major depressive disorder, bipolar disorder, anxiety disorder, schizophrenia, substance abuse disorder, alcohol dependence, hallucinogen persisting perception disorder, psychotic disorder, personality disorder, attention deficit hyperactivity disorder, obsessive compulsive disorder, and/or eating disorder*.

b *Classic psychedelics*.

c *Including cannabis, amphetamines, MDMA/ecstasy, cocaine, opiates, benzodiazepines, and/or ketamine*.

### Correlations

Bivariate Pearson correlations between primary and secondary well-being measures at baseline are shown in Table 3.

**Table 3:** Correlation matrix of main (primary and secondary) measures at baseline (time point 1; TP1).

		**WEMWBS**	**QIDS-SR_**16**_**	**RSE**	**LOT-R**	**TIPI-ES**	**MLQ-P**	**AAQ-II**	**BRS**	**CAMS-R**	**SCS**	**GQ-6**	**STS-U**	**SpREUK-SF-T**	**SCBCS**
WEMWBS	*r* *Sig*.	1	−0.64^**^ ** <0.001**	0.76^**^ ** <0.001**	0.64^**^ ** <0.001**	0.57^**^ ** <0.001**	0.55^**^ ** <0.001**	−0.65^**^ ** <0.001**	0.58^**^ ** <0.001**	0.62^**^ ** <0.001**	0.59^**^ ** <0.001**	0.56^**^ ** <0.001**	0.26^**^ <0.001	0.23^**^ <0.001	0.20^**^ <0.001
QIDS-SR_16_	*r* *Sig*.	−0.64^**^ ** <0.001**	1	−0.65^**^ <0.001	−0.54^**^ <0.001	−0.57^**^ <0.001	−0.35^**^ <0.001	0.62^**^ <0.001	−0.49^**^ <0.001	−0.48^**^ <0.001	−0.51^**^ <0.001	−0.39^**^ <0.001	−0.07 0.067	−0.08^*^ 0.048	0.02 0.553
RSE	*r* *Sig*.	0.76^**^ ** <0.001**	−0.65^**^ <0.001	1	0.70^**^ ** <0.001**	0.62^**^ <0.001	0.52^**^ <0.001	−0.69^**^ ** <0.001**	0.53^**^ <0.001	0.61^**^ <0.001	0.57^**^ <0.001	0.49^**^ <0.001	0.22^**^ <0.001	0.20^**^ <0.001	0.15^**^ <0.001
LOT-R	*r* *Sig*.	0.64^**^ ** <0.001**	−0.54^**^ <0.001	0.70^**^ ** <0.001**	1	0.56^**^ <0.001	0.42^**^ <0.001	−0.61^**^ <0.001	0.53^**^ <0.001	0.50^**^ <0.001	0.46^**^ <0.001	0.50^**^ <0.001	0.23^**^ <0.001	0.22^**^ <0.001	0.14^**^ <0.001
TIPI-ES	*r* *Sig*.	0.57^**^ ** <0.001**	−0.57^**^ <0.001	0.62^**^ <0.001	0.56^**^ <0.001	1	0.31^**^ <0.001	−0.67^**^ ** <0.001**	0.56^**^ <0.001	0.54^**^ <0.001	0.43^**^ <0.001	0.34^**^ <0.001	0.10^**^ 0.009	0.08^*^ 0.033	0.06 0.142
MLQ-P	*r* *Sig*.	0.55^**^ ** <0.001**	−0.35^**^ <0.001	0.52^**^ <0.001	0.42^**^ <0.001	0.31^**^ <0.001	1	−0.41^**^ <0.001	0.32^**^ <0.001	0.48^**^ <0.001	0.41^**^ <0.001	0.50^**^ <0.001	0.39^**^ <0.001	0.42^**^ <0.001	0.28^**^ <0.001
AAQ-II	*r* *Sig*.	−0.65^**^ ** <0.001**	0.62^**^ <0.001	−0.69^**^ ** <0.001**	−0.61^**^ <0.001	−0.67^**^ ** <0.001**	−0.41^**^ <0.001	1	−0.66^**^ ** <0.001**	−0.64^**^ <0.001	−0.53^**^ <0.001	−0.45^**^ <0.001	−0.10^*^ 0.011	−0.07 0.069	−0.07 0.069
BRS	*r* *Sig*.	0.58^**^ ** <0.001**	−0.49^**^ <0.001	0.53^**^ <0.001	0.53^**^ <0.001	0.56^**^ <0.001	0.32^**^ <0.001	−0.66^**^ ** <0.001**	1	0.53^**^ <0.001	0.41^**^ <0.001	0.37^**^ <0.001	0.12^**^ 0.003	0.08 0.054	0.11^**^ 0.004
CAMS-R	*r* *Sig*.	0.62^**^ ** <0.001**	−0.48^**^ <0.001	0.61^**^ <0.001	0.50^**^ <0.001	0.54^**^ <0.001	0.48^**^ <0.001	−0.64^**^ <0.001	0.53^**^ <0.001	1	0.43^**^ <0.001	0.41^**^ <0.001	0.23^**^ <0.001	0.20^**^ <0.001	0.16^**^ <0.001
SCS	*r* *Sig*.	0.59^**^ ** <0.001**	−0.51^**^ <0.001	0.57^**^ <0.001	0.46^**^ <0.001	0.43^**^ <0.001	0.41^**^ <0.001	−0.53^**^ <0.001	0.41^**^ <0.001	0.43^**^ <0.001	1	0.51^**^ <0.001	0.15^**^ <0.001	0.12^**^ 0.002	0.19^**^ <0.001
GQ-6	*r* *Sig*.	0.56^**^ ** <0.001**	−0.39^**^ <0.001	0.49^**^ <0.001	0.50^**^ <0.00	0.34^**^ <0.001	0.50^**^ <0.001	−0.45^**^ <0.001	0.37^**^ <0.001	0.41^**^ <0.001	0.51^**^ <0.001	1	0.36^**^ <0.001	0.30^**^ <0.001	0.36^**^ <0.001
STS-U	*r* *Sig*.	0.26^**^ <0.001	−0.07 0.067	0.22^**^ <0.001	0.23^**^ <0.001	0.10^**^ 0.009	0.39^**^ <0.001	−0.10^*^ 0.006	0.12^**^ 0.003	0.23^**^ <0.001	0.15^**^ <0.001	0.36^**^ <0.001	1	0.81^**^ ** <0.001**	0.43^**^ <0.001
SpREUK-SF-T	*r* *Sig*.	0.23^**^ <0.001	−0.08^*^ 0.048	0.20^**^ <0.001	0.22^**^ <0.001	0.08^*^ 0.033	0.42^**^ <0.001	−0.07 0.069	0.08 0.054	0.20^**^ <0.001	0.12^**^ 0.002	0.30^**^ <0.001	0.81^**^ **<0.001**	1	0.33^**^ <0.001
SCBCS	*r* *Sig*.	0.20^**^ <0.001	0.02 0.553	0.15^**^ <0.001	0.14^**^ <0.001	0.06 0.142	0.28^**^ <0.001	−0.07 0.069	0.11^**^ 0.004	0.16^**^ <0.001	0.19^**^ <0.001	0.36^**^ <0.001	0.43^**^ <0.001	0.33^**^ <0.001	1

*Each cell contains the Pearson correlation coefficient (r), followed by the p-value. For all measures, the sample size is N = 654. Cases are excluded pairwise. Correlations with WEMWBS (general well-being) are highlighted in dark grey. Non-significant correlations are provided in grey. Medium to large sized correlations with WEMWBS as well as the strongest 5 pairwise correlations are shown in bold. No corrections are done for multiple comparisons as these analyses were done for explorative purposes. For corrected values, see Supplementary Table 5*.

* *Correlation is significant at the 0.05 significance level (2-tailed)*.

** *Correlation is significant at the 0.01 significance level (2-tailed)*.

Results revealed that all secondary outcome measures correlated significantly with the primary outcome measure (WEMWBS), as was expected. Large positive correlations were found for WEMWBS and measures of self-esteem (RSE; *r* = 0.76, *p* < 0.0001), mindfulness (CAMS-R; *r* = 0.62, *p* < 0.0001), resilience (BRS; *r* = 0.58, *p* < 0.0001), personality subscale emotional stability (TIPI-ES; *r* = 0.57, *p* < 0.0001), gratitude (GQ-6; *r* = 0.56, *p* < 0.0001), and meaning in life subscale: “presence” (MLQ-P; *r* = 0.55, *p* < 0.0001). Large negative correlations were found with measures of psychological inflexibility (AAQ-II; *r* = −0.65, *p* < 0.0001) and depressive symptoms (QIDS-SR_16_, *r* = −0.64, *p* < 0.0001). Small correlations were found for the remaining measures.

The three largest covariances among the secondary measures included positive correlations between the two measures for spirituality (SpREUK-SF-T and STS-U; *r* = 0.81, *p* < 0.0001) and between optimism (LOT-R) and self-esteem (RSE; *r* = 0.70, *p* < 0.001), and a negative correlation between psychological inflexibility (AAQ-II) and self-esteem (RSE; *r* = 0.69, *p* < 0.0001). For Bonferroni corrected values, see Supplementary Table 5.

Explorative correlations of *change scores* between baseline and TP5 (4 weeks post) were also computed for all individual measures and are reported in Supplementary Table 4.

### GLM Repeated Measures MANOVA

#### Multivariate

Using Pillai's trace, the within-subject effect of time was found to be significant [*V* = 0.36, *F*_(28, 632)_ = 4.94, *p* < 0.0001, $\eta_{\text{p}}^{2}$ = 0.18], meaning that one or more of the included outcomes differed significantly between at least two timepoints.

#### Univariate

Repeated measures analyses of variance showed significant results for all main measures, except spirituality (both SpREUK-SF-T and STS-U) and compassion (SCBCS), see Table 4. The four largest univariate effect sizes were found for depressive symptoms, QIDS-SR_16_: *F*_(1.59, 260.03)_ = 53.64, *p* < 0.0001, $\eta_{\text{p}}^{2}$ = 0.25, optimism, LOT-R: *F*_(1.77, 290.88)_ = 26.22, *p* < 0.0001, $\eta_{\text{p}}^{2}$ = 0.14, self-esteem, RSE: *F*_(1.75, 286.64)_ = 25.01, *p* < 0.0001, $\eta_{\text{p}}^{2}$ = 0.13, and general mental well-being, WEMWBS: *F*_(1.82, 297.96)_ = 24.02, *p* < 0.0001, $\eta_{\text{p}}^{2}$ = 0.13. For a graphical depiction, see Supplementary Figure 1.

**Table 4:** Output Repeated Measures Multivariate Analysis of Variance (RM MANOVA), testing for statistical significance of post-experience change in relevant outcomes.

**Measure**	**RM MANOVA univariate test statistics**				**Descriptive statistics** ***M (SD)***		
	***df***	***F***	**Sig**.	**Partial η^2^**	**TP1 (1 week before^b^)**	**TP4 (2 weeks post^b^)**	**TP5 (4 weeks post^b^)**
WEMWBS	1.82, 297.96^a^	24.02	** <0.001**	**0.13**	48.86 (9.17)	52.30 (7.54)	51.79 (8.67)
QIDS-SR_16_	1.59, 260.03^a^	53.64	** <0.001**	**0.25**	6.18 (4.63)	4.01 (2.79)	3.82 (3.31)
RSE	1.75, 286.64^a^	25.01	** <0.001**	**0.13**	29.52 (6.37)	31.12 (5.93)	31.34 (5.97)
LOT-R	1.77, 290.88^a^	26.22	** <0.001**	**0.14**	21.02 (5.51)	22.43 (5.06)	22.51 (5.23)
TIPI-ES	2, 328	17.13	** <0.001**	**0.10**	9.27 (3.16)	9.92 (2.88)	10.12 (2.69)
MLQ-P	1.72, 282.47^a^	9.97	** <0.001**	**0.06**	21.47 (7.67)	23.18 (7.18)	22.73 (7.44)
AAQ-II	1.75, 286.15^a^	18.46	** <0.001**	**0.10**	32.13 (11.67)	29.92 (10.54)	29.22 (10.87)
BRS	2, 328	11.21	** <0.001**	**0.06**	3.28 (0.81)	3.42 (0.79)	3.46 (0.83)
CAMS-R	1.75, 287.48^a^	11.09	** <0.001**	**0.06**	32.88 (6.05)	33.88 (5.33)	34.33 (5.85)
SCS	1.86, 305.59^a^	3.24	**0.044**	**0.02**	33.67 (10.69)	34.97 (10.35)	35.21 (10.12)
GQ-6	2, 328	4.91	**0.008**	**0.03**	33.90 (6.06)	34.65 (5.64)	34.68 (6.13)
STS-U	2, 328	1.32	0.269	0.01	32.98 (8.15)	33.16 (8.11)	32.65 (7.95)
SpREUK-SF-T	1.86, 305^a^	0.85	0.421	0.01	11.12 (6.65)	11.30 (6.34)	11.07 (6.49)
SCBCS	1.87, 305.87^a^	1.33	0.267	0.01	4.67 (1.40)	4.68 (1.31)	4.59 (1.35)

a *Correction applied: Huyn Feldt (83)*.

b *With reference to the day the relevant psychedelic experience took place*.

*N = 165. Cases were excluded listwise. In bold the measures that were statistically significant at the 0.05 significance level (2-tailed)*.

### Exploratory Factor Analysis

Table 5 shows the pattern matrix with factor loadings after rotation, commonalities after extraction, output for assumption testing, and reliability measures. No assumptions were violated. The scree plot showed inflexions that would justify retaining either 2 or 3 factors. It was decided to retain 3 factors, consistent with Kaiser's criterion and aiming to better tease apart well-being. For the third factor, one scale (SCBCS) did not meet criteria for factor cross-loading and was therefore not included in subsequent calculation of the factor score.

**Table 5:** Relevant output Exploratory Factor Analysis (EFA); principal axis factoring.

**Extracted factors^a^**						
	**R-Matrix**				**Cronbach's** **α** **if deleted**	
**Factor name and included scales**	**Factor 1**	**Factor 2**	**Factor 3**	**Communalities post-factor extraction**	**Change scores (z)**	**Sum scores TP1 (z)**
**Being well (BW)**						
*Δ QIDS-SR_16_*	−0.80	0.08	0.21	0.50	0.75	0.87
*Δ RSE*	0.65	0.03	0.18	0.58	0.72	0.84
*Δ TIPI-ES*	0.55	0.09	−0.21	0.32	0.78	0.87
*Δ WEMWBS*	0.54	0.30	0.06	0.64	0.74	0.85
*Δ LOT-R*	0.53	−0.04	0.13	0.32	0.76	0.86
*Δ MLQ-P*	0.46	0.07	0.31	0.48	0.74	0.89
**Staying well (SW)**						
*Δ CAMS-R*	0.05	0.64	−0.02	0.44	0.55	0.67
*Δ AAQ-II*	−0.25	−0.53	0.03	0.50	0.53	0.63
*Δ SCS*	0.21	0.52	−0.11	0.39	0.55	0.66
*Δ BRS*	0.01	0.47	0.04	0.24	0.68	0.77
*Δ GQ-6*	−0.05	0.46	0.15	0.26	0.61	0.68
**Spirituality**						
*Δ STS-U*	−0.17	0.09	0.58	0.33	0.22	0.50
*Δ SpREUK-SF-T*	0.20	−0.17	0.44	0.21	0.32	0.60
*Δ SCBCS*	−0.26	0.29	0.30	0.17	0.40	0.89
Eigenvalues						
*Pre-factor extraction*	4.71	1.48	1.07			
*Post-factor extraction*	4.16	0.80	0.40			
Explained variance:						
*Pre-factor extraction*	33.63%	10.60%	7.64%	Cumulative: 51.87%		
*Post-factor extraction*	29.73%	5.73%	2.88%	Cumulative: 38.34%		
Cronbach's α, using:						
*Δ Scales (z)*	0.81	0.72	0.41			
*Sum scores scales TP1 (z)*	0.88	0.82	0.77			
**Factor analysis model statistics**						
KMO measure of sampling adequacy	0.89					
Bartlett's sphericity test		*χ^2^* *Sig*	690.38 <0.001			
Determinant |R|			0.021			
Reproduced correlations: non-redundant residuals			12.0%			

a *These three factors being factors of change implies that the measures included in each factor assemble together by means of their change; they change in a similar way over the selected timepoints*.

*N = 185. Factor loadings that together form a factor are highlighted in different shades of grey, as well as their corresponding reliabilities. Correlations lower than 0.3 are provided in grey. Change scores between TP1 and TP5 are used in the analyses and the change is indicated with “Δ.” Further, “(z)” indicates that scales were standardised to z-scores*.

After exploring the measures that loaded on each of the three resulting factors, factor 1 represented changes associated with “*Being well*,” factor 2 reflected changes associated with “*Staying well*” or maintaining wellness by reaching out to internal/external resources, and factor 3 reflected changes related to trait “*Spirituality*.”

Reliability analyses were carried out for the factors, both by using change scores (in line with the scores used in the EFA model) and scores at baseline. The former resulted in the following reliabilities for the three factors respectively: α = 0.81, α = 0.72, and α = 0.41 and the latter yielded reliabilities of: α = 0.88, α = 0.82, and α = 0.77.

### Factor Changes

**Figure 2:**
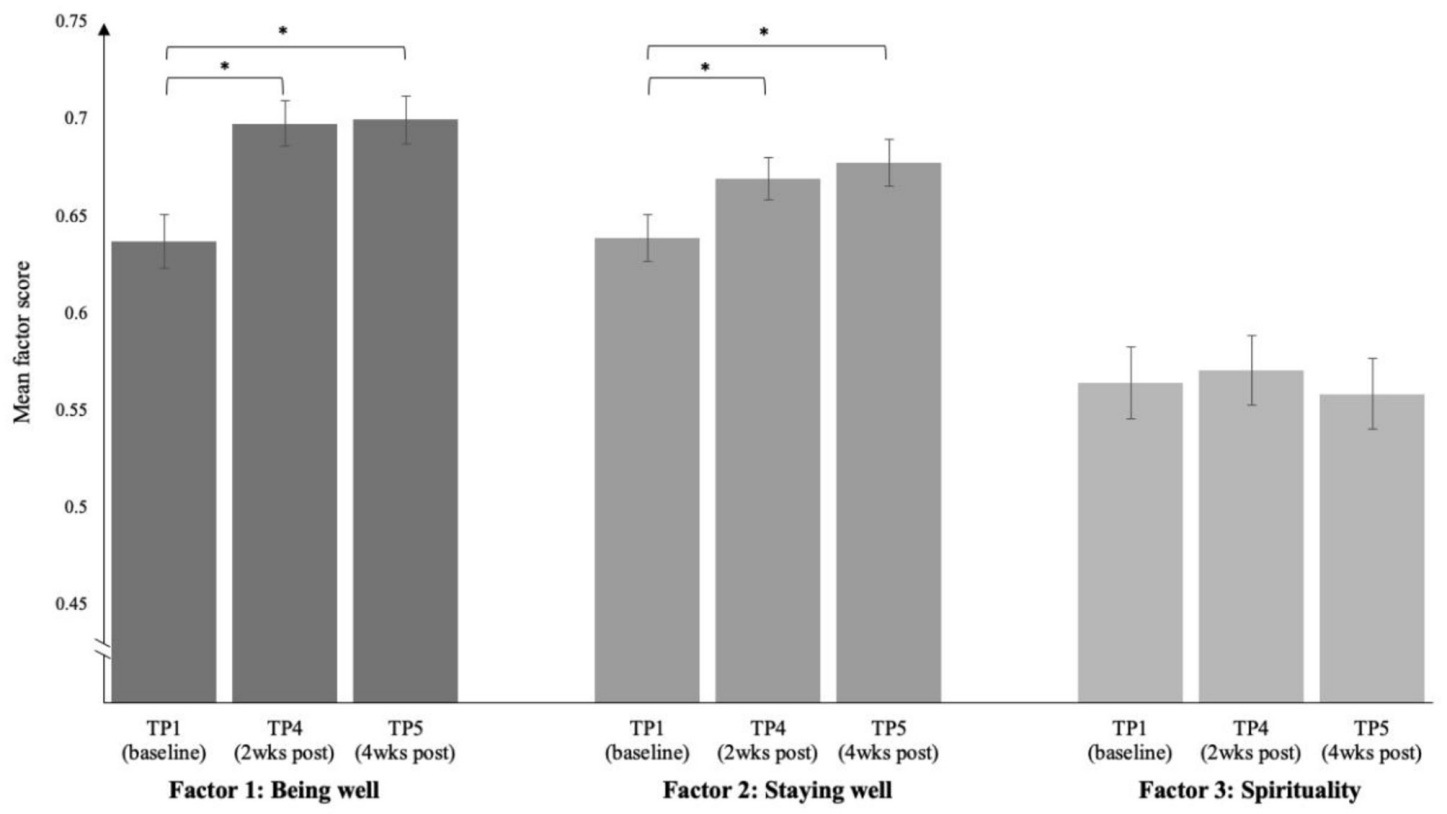
Per factor: factor scores changing over time (baseline, 2 weeks, and 4 weeks post-experience, respectively). Scores are normalised to allow for comparison between factors. Asterisks indicate a significant change over the specifically indicated change within time points, *p* < 0.05.

With three factors identified, it was subsequently assessed how they changed over time. Repeated measures multivariate analysis of variance (RM MANOVA) on the normalised sum scores of the identified factors *Being Well, Staying Well*, and *Spirituality* showed a significant within-subjects effect [*V* = 0.26, *F*_(6, 654)_ = 16.07, *p* < 0.0001, $\eta_{\text{p}}^{2}$ = 0.13]. Univariate tests showed a significant change for factors 1 and 2, with moderate effect sizes, but non-significant results for changes in factor 3 (see Table 6 and Figure 2).

**Table 6:** Relevant statistics for Repeated Measures Multivariate Analysis of Variance (RM MANOVA) with factor scores.

**Factor**	**RM MANOVA univariate test statistics**				**Descriptive statistics** ***M (SD) (normalised factor scores)***		
	***df***	***F***	**Sig**.	**Partial η^2^**	**TP1 (1 week before^b^)**	**TP4 (2 weeks post^b^)**	**TP5 (4 weeks post^b^)**
1: Being well	1.61, 264.41	48.74^a^	** <0.001**	**0.23**	0.64 (0.18)	0.70 (0.15)	0.70 (0.16)
2: Staying well	1.88, 307.81	18.33^a^	** <0.001**	**0.10**	0.64 (0.16)	0.67 (0.14)	0.68 (0.15)
3: Spirituality	1.92, 314.26	1.64^a^	0.196	0.01	0.56 (0.24)	0.57 (0.23)	0.56 (0.23)

a *Estimate of sphericity (ε) >0.75; Huyn Feldt correction applied (83)*.

b *With reference to the day relevant psychedelic experience took place*.

*N = 165. Cases were excluded listwise. In bold measures that were statistically significant at the 0.05 significance level*.

Pairwise comparisons using Bonferroni correction revealed significant changes between TP1 and TP4 as well as between TP1 and TP5 for factor 1 and 2 (both *p* < 0.0001)—in line with hypotheses. Factor 1 followed a quadratic trend (*p* < 0.0001, $\eta_{\text{p}}^{2}$ = 0.17), factor 2 a primary linear (*p* < 0.0001, $\eta_{\text{p}}^{2}$ = 0.15) and slight quadratic trend (*p* = 0.042, $\eta_{\text{p}}^{2}$ = 0.03).

### Follow-Up: TP6 Data

Approximately 2 years post-experience, *N* = 64 respondents from the original baseline sample filled out the questionnaires again. Changes in the identified factors were now assessed including the additional time point.

#### Change Over Time

**Figure 3:**
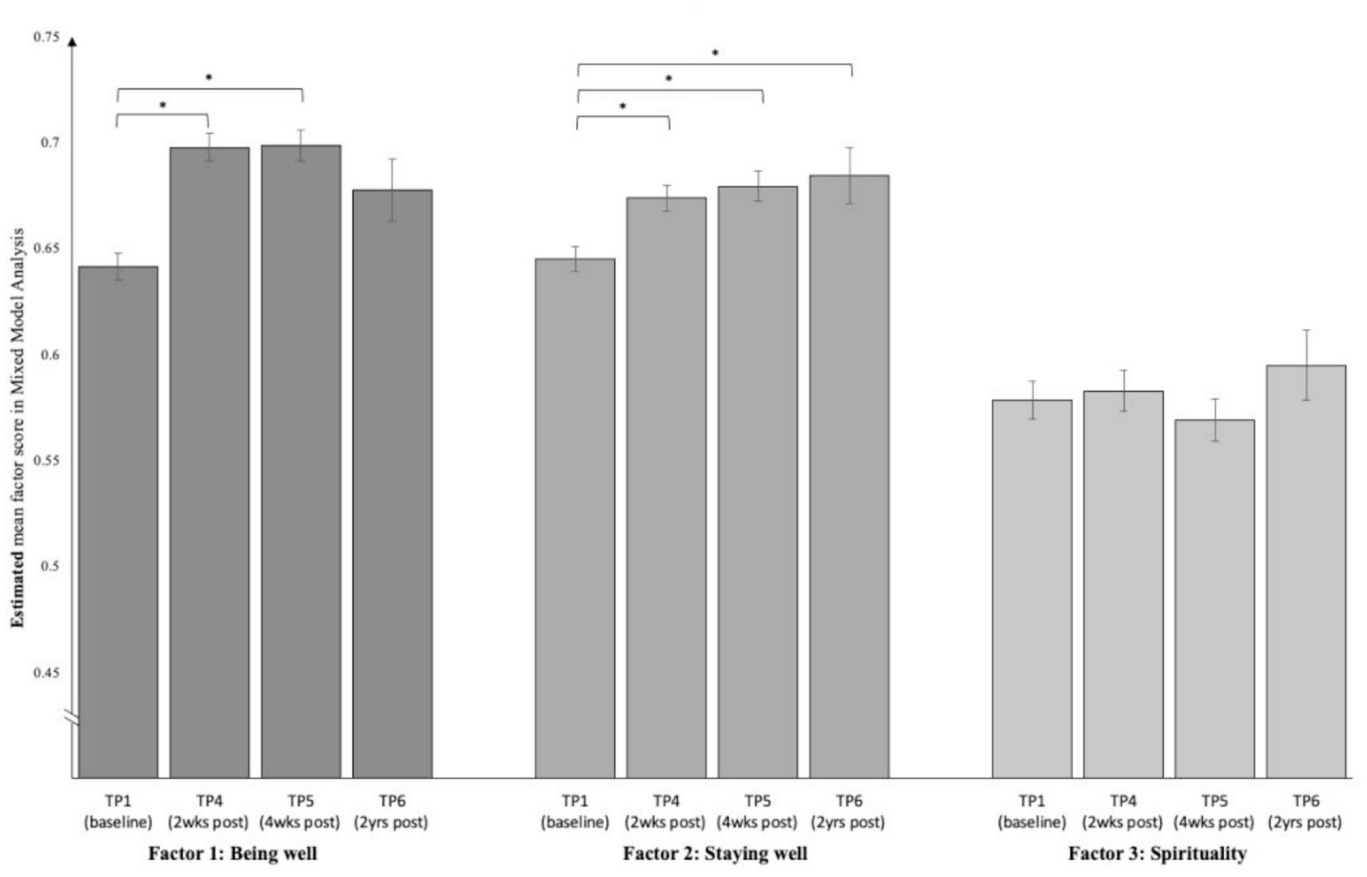
Estimated means and standard errors across the four time points (TPs), based on Mixed Model analyses. *N* = 698. Asterisks indicate a significant difference from zero for the fixed effect parameter estimates *t*-test, *p* < 0.05.

Linear Mixed Model analyses showed parameter estimates that implied a sustained increase in *Being Well* and, particularly, *Staying Well* over all four time points. That is, the aforementioned increase that was found from baseline to 2 and 4 weeks post-psychedelic, now also seemed to prolong to TP6, 2 years later, see Table 7 and Figure 3. *Post-hoc* pairwise comparisons (also in Table 7) indicated that baseline scores differed significantly from all other three timepoints for *Staying Well* but fell to trend-level (*p* = 0.08) for *Being Well* at TP6.

**Table 7:** Relevant outcomes Mixed Model analyses with factor scores across the four timepoints (TPs).

			**Factor 1: Being well**	**Factor 2: Staying well**	**Factor 3: Spirituality**
Model	Covariance structure		Unstructured	Unstructured	Unstructured
	Number of parameters		15	15	15
	Number of subjects incl.		698	698	698
Information criterion	Akaike's (AIC)		−1690.50	−1865.48	−1131.87
Parameter Estimates of fixed effects (E) + matching significance (p)	Intercept		**E** **=** **0.641;** ***p*** **≤** **0.001**	**E** **=** **0.645;** ***p*** **≤** **0.001**	**E** **=** **0.578;** ***p*** **≤** **0.001**
	TP 1: baseline (reference)		–	–	–
	TP4: 2 weeks after		**E** **=** **0.056;** ***p*** **≤** **0.001**	**E** **=** **0.029;** ***p*** **≤** **0.001**	E = 0.004; *p = 0.4*26
	TP5: 4 weeks after		**E** **=** **0.057;** ***p*** **≤** **0.001**	**E** **=** **0.034;** ***p*** **≤** **0.001**	E = −0.009; *p = 0.0*90
	TP6: 2 years after		**E** **=** **0.037;** ***p=*** **0.014**	**E** **=** **0.039;** ***p=*** **0.004**	E = 0.016; *p = 0.2*80
Pairwise comparisons^*^	Baseline (TP1)	TP4	***p*** **≤** **0.001**	***p*** **≤** **0.001**	*p =* 1.00
		TP5	***p*** **≤** **0.001**	***p*** **≤** **0.001**	*p =* 0.541
		TP6	*p =* 0.084	***p=*** **0.023**	*p =* 1.00
Estimated means and standard errors	TP1		*M* = 0.641; *SE* = 0.009	*M* = 0.645; *SE* = 0.006	*M* = 0.579; *SE* = 0.047
	TP4		*M* = 0.698; *SE* = 0.009	*M* = 0.674; *SE* = 0.006	*M* = 0.583; *SE* = 0.047
	TP5		*M* = 0.699; *SE* = 0.009	*M* = 0.680; *SE* = 0.007	*M* = 0.569; *SE* = 0.047
	TP6		*M* = 0.678; *SE* = 0.016	*M* = 0.685; *SE* = 0.013	*M* = 0.595; *SE* = 0.049

*Note. In bold parameter estimates that were statistically significant at the 0.05 significance level*.

* *Adjustment for multiple comparisons: Bonferroni*.

#### Attrition (Bias)

**Figure 4:**
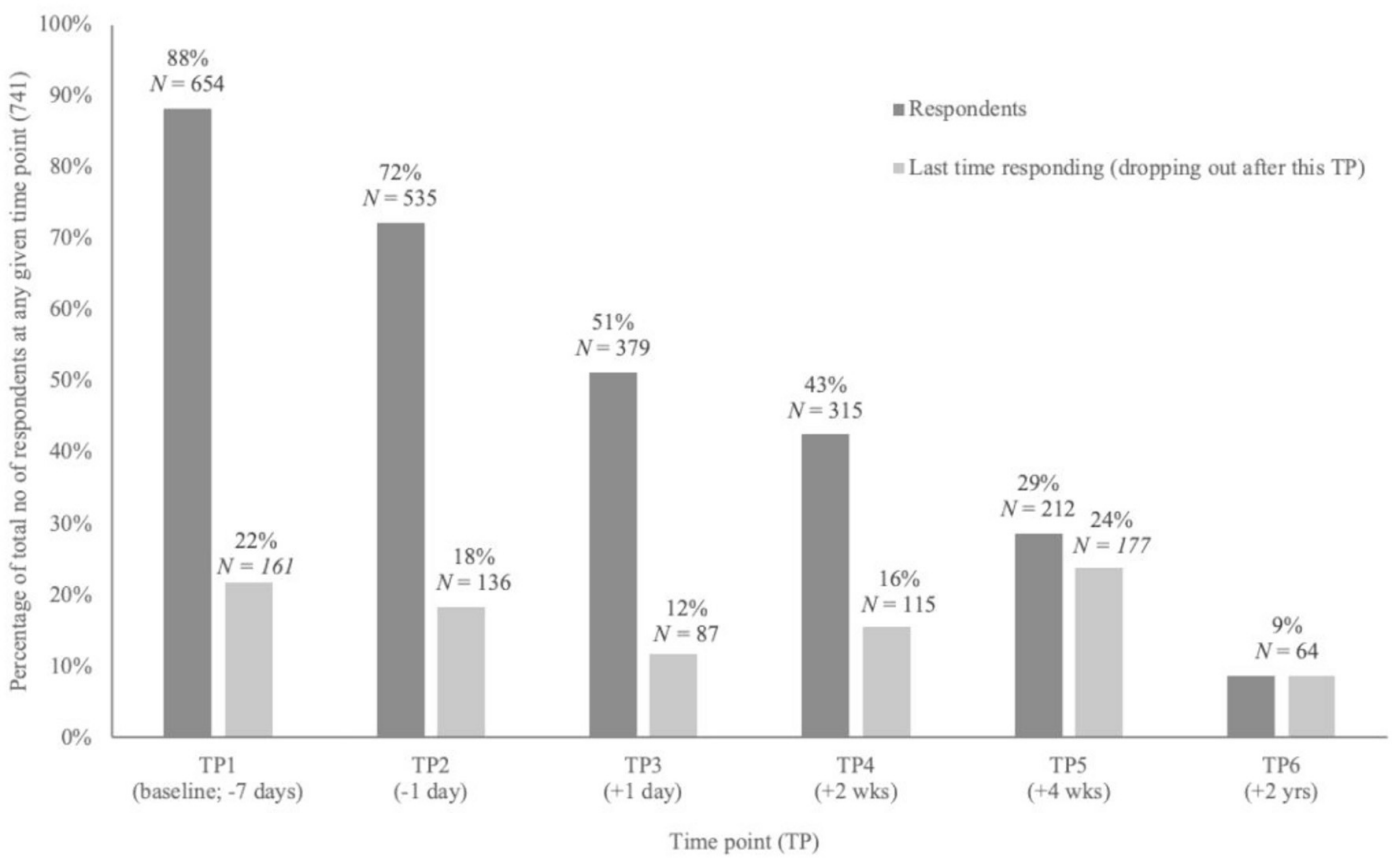
Attrition. Per time point the sample size and number of respondents who did not complete any more surveys after this particular time point (dropping out).

Out of a total of 741 participants responding to any timepoint of the study, 677 (91.4%) dropped out at, or prior to, the final follow-up survey at 2 years post-experience. More specifically, Figure 4 shows the total sample sizes as well as the attrition rates for each time point, in reference to the total (100%) number of respondents at any given time point. In total, 32 (4.3%) respondents completed all six time points.

To test for potential attrition biases in this sample, a Multivariate Analysis of Variance was run and showed no significant difference between TP6 completers and non-completers on the selected factor change scores (TP1 to TP5) and two of the three subjective measures (TP3). That is, TP6 completers and non-completers did not show a different change from baseline to 4 weeks after the experience on *Being Well* or *Staying Well*, nor were significant differences found for the subjective measures EBI, MEQ and CEQ.

For a related, more comprehensive, exploratory analysis of attrition, see Hübner et al. (84).

## Discussion

The aim of the present study was to assess the effects of psychedelics on mental well-being in an opportunity sample, using a broad range of measures. Results supported our main prior hypothesis that psychedelic experiences lead to broad increases in well-being in those that have a prior intention to use a psychedelic compound, and well-being scores remained elevated 2 years after the experience.

Scores on both identified factors *Being Well* and *Staying Well* were significantly increased post-psychedelic use. These factors can be seen as reflecting: (1) a current state of being well, e.g., higher trait optimism, positive affect, and self-esteem; and (2) a more prospective staying well factor, which covers attributes such as resilience, psychological flexibility, and mindfulness—three constructs that are found to be inter-related and contribute to positive mental health (13, 68, 69, 85). In line with this, the current study found these phenomena *changing together* after a psychedelic experience.

These findings lend support to the view that psychedelics have a general positive effect on well-being; promoting psychological wellness and resilience in the medium to long-term. Considering the current magnitude of the burden of mental illness, its increasing prevalence, and the growing costs of healthcare associated with mental illness, promotion and longer-term protection of well-being (“staying well”) is considered a priority area and could yield personal, social, and economical benefits (5, 9). Since the data for the current study were collected in a naturalistic and observational manner, they have good ecological validity, bearing relevance to the apparently increasing prevalence of use of psychedelics in the west (86)—a trend that seems likely to continue. The finding that scores on the *Staying Well* factor were increased at all three follow-up time points, also hints at psychedelics' prophylactic potential (40), bearing relevance to a current rethinking in mental healthcare toward proactive (preventive), rather than reactive interventions (10, 11).

Mindfulness-based and positive psychology approaches are presently being explored as an early intervention strategy, with apparent success in improving well-being by promoting affect tolerance and resilience (i.e., the ability to bounce back or recover from stress) through accepting and by decreasing maladaptive coping strategies, such as experiential avoidance, in response to stress (13, 87–90). Here, an interesting parallel can be seen, i.e., between the improvement of mindfulness capacities—in clinical as well as non-clinical samples—through meditation and therapeutic interventions, and through psychedelic experiences, as current findings and previous studies show (36–38, 91, 92). Furthermore, acute exposure to psychedelics may be followed by a stage of increased psychological acceptance, offering a window of opportunity for psychotherapeutic gains (92). “Third wave” psychotherapies, such as Acceptance and Commitment Therapy (ACT), may be particularly relevant here (93, 94). Hence, rather than looking at behavioural interventions and the application of psychedelics as separate pathways of achieving a similar goal, it is suggested that they may be harmonised (95–97). Traditional methods for training mindfulness skills may be complemented directly or indirectly via psychedelic use or therapy, aiming to protect well-being.

The feasibility of early intervention in mental health is being increasingly discussed (98). Given the special burden of chronic, mental, and physical illness predicted by early-life suffering (5, 6, 99) and the particular limitations of psychiatric drug interventions in young people (100), there is a need for safer, more effective early intervention strategies. As always, the merits of early intervention need to be factored against the specific risks associated with a given intervention—particularly if: (1) no pathology in present (5), and (2) the population is young and vulnerable.

The present study identified a specific factor containing validated scales pertaining to “Staying Well.” Scores on this factor were significantly increased at 2 and 4 weeks after the psychedelic experience and remained significantly elevated at 2 years. This finding is particularly intriguing, as it has implications for the long-term positive psychological effects of psychedelics, as well as their use as early intervention and/or as prophylactic tools. Used with care, psychedelics may have potential to complement early intervention or prophylactic strategies, e.g., using low-dose psychedelic therapy to improve receptivity to, and enhance the action of, mindfulness-based practises designed for this purpose. In this regard, it is worth noting that exposure to psychedelic plant medicines among the young is endorsed by certain cultures e.g., as part of coming of age ceremonies (101–103) or religious ceremonies (104, 105). Taken together, these findings strongly warrant further research on the mental health effects of psychedelic use in adolescents and young adults, specifically in relation to their hypothesised capacity to foster resilience and other protective psychological capacities.

Despite this promise, we recognise that historical negative stigma surrounding psychedelics may make it particularly difficult to develop an early intervention trial. Indeed, it be prudent to recognise that even if the risk of severe iatrogenesis via psychedelics is very rare, if it were to happen, the personal, familial and perhaps even broader political impact would be considerable. This consideration is particularly pertinent when intervening in developing minds and brains where no existing pathology is evident. Equally, however, such caution must be weighed against the potential for long-term psychological benefit at the aggregate level and a commitment to scientific process, particularly when evidence suggests that testing of a novel hypothesis is worthwhile and could ultimately bring important benefits.

A key question for the present study was: which of the several measures associated with well-being are most sensitive to change? This question is important, not least because of considerations of efficiency in the design of future studies, where, for example, the number of questionnaires could be reduced to just a few, sensitive but sufficiently orthogonal and therefore complementary ones. This issue is particularly relevant in prospective web-based surveys where high attrition rates due to participant burden are a common problem (84, 106).

Results revealed that the most sensitive measures were those that were classified under the *Being well* factor. More specifically, the three largest changes were seen in QIDS-SR_16_ (depressive symptoms), TIPI-ES (emotional stability), and WEMWBS (general mental well-being) scores, where effect sizes were large. This implies that these are particularly sensitive measures that can be usefully employed in future studies.

We are mindful, however, that our statistical approach involved factorising questionnaires according to how scores *changed* after a psychedelic. This approach could be critiqued, as it does not reflect whether the constructs that the scales are intended to measure are intrinsically distinct from each other, e.g., as might be demonstrated if scores from only one particular timepoint were entered into the factor analysis. It rather reflects the correlation of changes in scores on those measures. This approach was explorative and should therefore not predominantly guide the decision for future scales to include. As shown in Table 3, there do exist strong correlations between the different measures of well-being when looking at scores on one single time point (baseline), but pairwise relationships were not uniform in strength and some scales were inversely related. We believe that using the diagnostically validated QIDS-SR_16_ to measure depression severity is complementary to measuring general well-being with the WEMWBS and thus advocate using these two brief measures in future studies.

The five scales loading onto *Staying Well* were generally less sensitive to change than the six *Being Well* questionnaires. However, since they were found to be distinct from the *Being Well* measures, they can yield additional information. Selecting which *Staying Well* scales to include in future studies may best be informed by psychological framework preferences e.g., Acceptance and Commitment Therapy (ACT) and the AAQ-II vs. Mindfulness-Based Cognitive Therapy (MB-CT) and the CAMS-R. We are aware that some have critiqued the AAQ-II, however (107–109), and the CAMS-R loaded more strongly onto the *Staying Well* factor and not at all onto the *Being Well* one. Thus, the CAMS-R may be more the more useful of these two when combined with the QIDS-SR_16_ and WEMWBS. If one was to place special value on efficiency and framework neutrality however, the BRS might be a good choice, as it contains only six items, indexes general resilience, and, like the CAMS-R, showed a good factor preference for *Staying Well* vs. *Being Well*.

The third factor, *Spirituality*, contained three measures that index phenomena not universally regarded as relevant to well-being (Supplementary Table 1). Specifically, the SpREUK-SF-T is intended to measure trust in divine forces, the STS-U refers to belief in unity or interconnectedness, and the SCBCS enquires about feelings of compassion. These measures were less sensitive to change after psychedelic use in the current sample. This could be a culture and context dependent result, given that the sample was predominantly Western, and participants may not have taken the psychedelic with a spiritual intention in mind or been in a (e.g., ceremonial) context where spiritual themes were welcomed or promoted. Future studies and analyses could examine the influence of contextual factors on such outcomes, with the hypothesis that they do indeed have a significant influence (42). Another explanation for this negative finding could be that the here used *Spirituality* measures are trait-based, i.e., the SpREUK-SF-T and STS-U do not enquire about spiritual experiences *per se*, but rather enquire how one generally perceives oneself; as such, they may be less sensitive to the effects of psychedelics.

There are some important limitations to this study. A major one is the lack of experimental control, which meant that we could not verify any of the reported information and thus had to take the validity of responses on faith, including those relating to drug usage, purity and dosage. Haijen et al. (32) mention this limitation as well and list some variables important to acknowledge in future studies, regarding safe and effective preparation for, and mediation of, psychedelic experiences. Neither were we able to control for expectancy in any way, a major potential source of bias, particularly in young people (110–112).

Relatedly, there was a significant risk of confirmation bias in this study; the sample consisted of people intending to take a psychedelic through their own initiative and many reported previous experience with psychedelic drugs (90.5%), as well as a generally positive stance toward the (therapeutic) potential of psychedelics (32). Participants were also predominantly male (74.2%), employed or a student (90.3%), and western (i.e., 50% USA or UK), which limits generalisability and extrapolations beyond this population. These and other limitations are elaborated on Haijen et al. (32). Furthermore, the attrition analysis conducted in the current study was also intended to scrutinise the nature of the sample.

Another potential bias may have occurred via the high attrition rate: i.e., there might have been a skew toward positive findings if drop-outs occurred in those who did not respond well. To assess this possibility, we explored the question: did people who dropped out before the final follow-up survey at 2 years (TP6) show a more negative trajectory of change in *Being Well* and *Staying Well* than those 64 people completing that final survey? Also, did differences exist in scores on subjective acute (TP3) measures, such as the intensity of challenging experiences, in those who dropped out? Results were reassuring in terms of potential attrition bias; participants experiencing a more challenging experience under the psychedelic, or a more negative trajectory in their change in well-being scores, were not more likely to drop-out.

Furthermore, this study population was heterogeneous, i.e., different substances, set and setting, and also a high distribution of lifetime mental illness diagnoses. In future studies, exclusion criteria could be extended, encouraging safe use e.g., by screening more carefully on psychosis and related disorders. In case a similar opportunity sample was included in future studies, it would be interesting to study response differences in people *with* vs. *without* psychiatric history, or *with* vs. *without* subsequent psychedelic experiences. For the latter, questions on drug use should be added to the follow-up surveys. Lastly, interesting for future research may be to look at predictors, such as intentions, in the factors of change that were found in the current study.

In conclusion, the present study assessed changes in several complementary facets of mental well-being following a psychedelic experience. In line with prior hypotheses, comprehensive positive changes in well-being were observed. Three major components of change were identified: “Being well,” “Staying well,” and “Spirituality.” The first two increased significantly at 2 and 4 weeks after the relevant psychedelic experience, with *Staying Well* remaining elevated at 2-year follow-up. These findings support the view that psychedelic use can both promote and protect psychological wellness. The findings should inspire more controlled research into the impact of psychedelics on mental health in healthy populations. Longitudinal studies in young populations may have special value, with potential implications for the prophylactic value of psychedelic therapy.

## Data Availability Statement

Datasets are available on request: The raw data supporting the conclusions of this article will be made available by the authors, without undue reservation.

## Ethics Statement

The studies involving human participants were reviewed and approved by Joint Research Compliance Office Imperial College London. The patients/participants provided their written informed consent to participate in this study.

## Author's Note

We are currently running new online survey studies. For more information, see www.psychedelicsurvey.com.

## Author Contributions

KM wrote this paper. RC-H and HK edited it with feedback from MK, EH, and DE. All authors read and approved the final manuscript.

## Conflict of Interest

The authors declare that the research was conducted in the absence of any commercial or financial relationships that could be construed as a potential conflict of interest.

## Footnotes

^1^Sometimes also referred to as “positive mental health” or “flourishing.” From here on shortened as “well-being.”

^2^From here on shortened as “psychedelics.”
