# Supplementary material for: Sustained, Multifaceted Improvements in Mental Well-Being Following Psychedelic Experiences in a Prospective Opportunity Sample
Source: Front Psychiatry. 2021 Jun 29;12:647909. doi: 10.3389/fpsyt.2021.647909 (PMC8277190; doi:10.3389/fpsyt.2021.647909)
Supplement: Supplementary file 1 [file Table_1.docx]

| Supplementary Material: Table 1. *Definitions and approaches to the construct of mental well-being.*  **Mental well-being** | | | | | | | | |
| --- | --- | --- | --- | --- | --- | --- | --- | --- |
|  |  | **Hedonia**  Feeling good | | |  | **Eudaimonia**  Functioning well | | |
| Also called |  | “Subjective well-being” | | |  | “Psychological well-being” | | |
| Relation to happiness *(Disabato et al., 2016)*^]^: |  | Pleasure-orientation of happiness. | | |  | Meaning-orientation of happiness, emphasising purpose in life and goal-directed behaviour in order to reach one’s full potential. | | |
| Theoretical models focusing on hedonia, eudaimonia, or both. |  | *Diener  (1984)* | | *Kahneman*  *(1999*) |  | *Antonovsky (1987)* | *Ryff*  *(1989)* | *Ryan & Deci (2001)* |
|  |  | Happiness, positive mood (affect) and life satisfaction | | Pleasure attainment (maximize positive affect) and pain avoidance (minimize negative affect) |  | Comprehen-sibility  Manageability  Meaningfulness | Autonomy  Environmental Mastery  Personal growth  Purpose in life  Positive Relationships  Self-acceptance | Autonomy  Competence  Relatedness |
|  |  |  |  | |  |  |  |  |
|  |  | *Seligman (2011) -* PERMA model of “Positive mental health”: | | | | | | |
|  |  | Positive Emotion, Engagement, Relationships, Meaning, and Accomplishment. | | | | | | |
|  |  | *Huppert & So (2013)* – Multidimensional construct “Flourishing”: | | | | | | |
|  |  | Competence, Emotional stability, Engagement, Meaning, Optimism, Positive emotion, Positive relationships, Resilience, Self-esteem, and Vitality. | | | | | | |
| *Note*. Only a selection of theoretical models is included. | | | | | | | | |
