# Supplementary material for: Sustained, Multifaceted Improvements in Mental Well-Being Following Psychedelic Experiences in a Prospective Opportunity Sample
Source: Front Psychiatry. 2021 Jun 29;12:647909. doi: 10.3389/fpsyt.2021.647909 (PMC8277190; doi:10.3389/fpsyt.2021.647909)
Supplement: Supplementary file 2 [file Table_2.docx]

| Supplementary Material: Table 2.  *Drug variables, collected at the third time point (TP3).* | | |
| --- | --- | --- |
| **Total** | | **N = 397** |
| Drug type | LSD/1P-LSD | 183 (48,3%) |
|  | Psilocybin | 109 (28.8%) |
|  | Ayahuasca | 42 (11.1%) |
|  | DMT/5-MeO-DMT | 12 (3.2%) |
|  | Mescaline (Peyote, San Pedro) | 10 (2.8%) |
|  | Salvia divinorum | 0 (0%) |
|  | Iboga/ibogaine | 0 (0%) |
|  | Other | 23 (6.1%) |
| Drug dose^a^ | A low dose | 39 (10.3%) |
|  | A moderate dose | 144 (38.0%) |
|  | A high dose | 135 (35.6%) |
|  | A very high dose | 37 (9.8%) |
|  | An extremely high dose | 24 (6.3%) |
| *Note.* Absolute frequencies and the percentages corresponding to the absolute frequencies are shown.  ^a^ Drug dose was reported in LSD equivalents: no more than 50 micrograms of LSD, no more than 100 micrograms of LSD, no more than 200 micrograms of LSD, no more than 300 micrograms of LSD, and more than 300 micrograms of LSD, respectively. | | |
