# Supplementary material for: Sustained, Multifaceted Improvements in Mental Well-Being Following Psychedelic Experiences in a Prospective Opportunity Sample
Source: Front Psychiatry. 2021 Jun 29;12:647909. doi: 10.3389/fpsyt.2021.647909 (PMC8277190; doi:10.3389/fpsyt.2021.647909)
Supplement: Supplementary file 3 [file Table_3.docx]

| Supplementary Material: Table 3.  *Overview of all included outcome measures and some of their characteristics (extended version of Table 1 in-text)* | | | | | |
| --- | --- | --- | --- | --- | --- |
|  | **Measure** | **Construct measured and subscale(s) used**  (if present) | **No of items^a^** | **Example item** | **Measurement scale^c^** |
| **Primary measure** | **WEMWBS**  The Warwick-Edinburgh Mental Well-being scale (Tennant et al., 2007) | **Mental well-being** | 14 | I've been feeling optimistic about the future. | 5-point Likert scale: ‘None of the time’ to ‘All of the time’. |
| **Secondary measures** | **QIDS-SR_16_**  Quick Inventory of Depression Symptoms (Rush et al., 2003) | **Depression symptoms**  Items on sleep, sad, appetite, concentration, sleep, view of oneself, suicide, interest, energy, psychomotor. | 16 | General interest: ^b^  There is no change from usual in how interested I am in other people or activities.  I notice that I am less interested in people or activities.  I find I have interest in only one or two of my formerly pursued activities.  I have virtually no interest in formerly pursued activities. | 4 Answer options linked to a given symptom or phenomenon. |
|  | **RSE**  Rosenberg Self-Esteem Scale (Rosenberg, 1965) | **Self-esteem** | 10 | I feel that I'm a person of worth, at least on an equal plane with others. | 4-point Likert scale: ‘Strongly agree’ to ‘Strongly disagree’. |
|  | **LOT-R**  Revised Life Orientation Test (Glaesmer et al., 2012) | **Optimism**  Both optimism and pessimism (reversed) items included, filler items excluded in analysis. | 10 | In uncertain times, I usually expect the best. | 5-point Likert scale: ‘I agree a lot’ to ‘I disagree a lot’. |
|  | **TIPI**  Ten-item Personality Inventory (Gosling, Rentfrow, & Swann, 2003) | **Personality**  1/5 Subscales: Emotional stability (**TIPI-ES**) | 2 | I see myself as: anxious, easily upset. | 7-point Likert scale: ‘Disagree strongly’ to ‘Agree strongly’. |
|  | **MLQ**  Meaning in Life Questionnaire (Steger, Kashdan, Sullivan, & Lorentz, 2008) | **Meaning in life**  1/2 subscales: Presence (**MLQ-P**). | 5 | My life has a clear sense of purpose. | 7-point Likert scale ranging from ‘Absolutely untrue’ to ‘Absolutely true’. |
|  | **AAQ-II**  Acceptance and Action Questionnaire-II (Bond et al., 2011) | **Psychological inflexibility and experiential avoidance** | 10 | My thoughts and feelings do not get in the way of how I want to live my life. (reversed item) | 7-point Likert scale: ‘Never true’ to ‘Always true’. |
|  | **BRS**  Brief Resilience Scale (Smith et al., 2008) | **Resilience** | 6 | It does not take me long to recover from a stressful event. | 5-point Likert scale: ‘Strongly disagree’ to ‘Strongly agree’. |
|  | **CAMS-R**  Revised Cognitive and Affective Mindfulness Scale (Feldman, Hayes, Kumar, Greeson, & Laurenceau, 2007) | **Mindfulness** | 12 | I can usually describe how I feel at the moment in considerable detail. | 4-point Likert scale: ‘Rarely/not at all’ to ‘Almost always’. |
|  | **SCS**  Social Connectedness Scale (Lee & Robbins, 1995) | **Social connectedness** | 8 | Even around people I know, I don't feel that I really belong. | 6-point Visual Analogue Scale: ‘Strongly agree’ to ‘Strongly Disagree’ |
|  | **GQ-6**  Gratitude Questionnaire (McCullough, Emmons, & Tsang, 2002) | **Gratitude** | 6 | If I had to list all the things I felt grateful for, it would be a very long list. | 7-point Likert scale: ‘Strongly disagree’ to ‘Strongly agree’. |
|  | **STS**  Spiritual Transcendence Scale (Piedmont, 1999) | **Spirituality**  1/3 Subscales: Universality (**STS-U**): belief of unity and purpose of life. | 9 | Although individual people may be difficult, I feel an emotional bond with all of humanity. | 5-point Likert scale: ‘Strongly disagree’ to ‘Strongly agree’. |
|  | **SpREUK-SF**  Spiritual and Religious Attitudes in Dealing with Illness – modified short form (Büssing, Ostermann, & Matthiessen, 2005) | **Spirituality**  1/5 Subscale: Trust (**SpREUK-SF-T**): trust in external guidance. | 6 | I trust in a higher power that carries me through. | 5-point Likert scale ranging from ‘Does not apply at all’ to ‘Definitely applies’. |
|  | **SCBCS**  Santa Clara Brief Compassion Scale (Hwang et al., 2008) | **Compassion** | 5 | When I hear about someone (a stranger) going through a difficult time, I feel a great deal of compassion for him or her. | 7-point Visual Analogue Scale ranging from ‘Strongly agree’ to ‘Strongly Disagree’. |
| ^a^ Number of items included in the analyses, i.e. only those of selected (sub)scales.  ^b^ All outcome measures are continuous (interval or ratio) measures. | | | | | |
