# Supplementary material for: Sustained, Multifaceted Improvements in Mental Well-Being Following Psychedelic Experiences in a Prospective Opportunity Sample
Source: Front Psychiatry. 2021 Jun 29;12:647909. doi: 10.3389/fpsyt.2021.647909 (PMC8277190; doi:10.3389/fpsyt.2021.647909)
Supplement: Supplementary file 4 [file Table_4.docx]

| Supplementary Material: Table 4.  *Correlation matrix of change scores between Timepoint 1 and Timepoint 5 (change 15) for the main measures.* | | | | | | | | | | | | | | | |
| --- | --- | --- | --- | --- | --- | --- | --- | --- | --- | --- | --- | --- | --- | --- | --- |
|  |  | WEMWBS change15 | QIDS change 15 | RSE change 15 | LOT-R change 15 | TIPI-ES change 15 | MLQ-P change 15 | AAQ-II change 15 | BRS change 15 | CAMS-R change 15 | SCS change 15 | GQ-6 change 15 | STS-U change 15 | SpREUK-SF-T change 15 | SCBCS change 15 |
| WEMWBS change 15 | r  Sig. | 1 | **-.49^**^**  **<.001** | **.56^**^**  **<.001** | **.41^**^**  **<.001** | **.40^**^**  **<.001** | **.54^**^**  **<.001** | **-.54^**^**  **<.001** | **.36^**^**  **<.001** | **.48^**^**  **<.001** | **.47^**^**  **<.001** | **.31^**^**  **<.001** | **.21^**^**  **.003** | **.21^**^**  **.005** | .06  .442 |
| QIDS-SR_16_  change 15 | r  Sig. | **-.49^**^**  **<.001** | 1 | **-.48^**^**  **<.001** | -.36^**^  <.001 | -.36^**^  <.001 | -.32^**^  <.001 | .34^**^  <.001 | -.13  .075 | -.30^**^  <.001 | -.32^**^  <.001 | -.15^*^  .044 | .03  .661 | -.08  .268 | .09  .222 |
| RSE  change 15 | r  Sig. | **.56^**^**  **<.001** | **-.48^**^**  **<.001** | 1 | .41^**^  <.001 | .36^**^  <.001 | **.54^**^**  **<.001** | **-.46^**^**  **<.001** | .28^**^  <.001 | .40^**^  <.001 | .40^**^  <.001 | .28^**^  <.001 | .16^*^  .032 | .25^**^  .001 | .13  .070 |
| LOT-R  change 15 | r  Sig. | **.41^**^**  **<.001** | -.36^**^  <.001 | .41^**^  <.001 | 1 | .28^**^  <.001 | .42^**^  <.001 | -.35^**^  <.001 | .16^*^  .031 | .25^**^  .001 | .32^**^  <.001 | .16^*^  .029 | .10  .195 | .17^*^  .023 | .14  .059 |
| TIPI-ES change 15 | r  Sig. | **.40^**^**  **<.001** | -.36^**^  <.001 | .36^**^  <.001 | .28^**^  <.001 | 1 | .25^**^  <.001 | -.37^**^  <.001 | .22^**^  .003 | .20^**^  .007 | .29^**^  <.001 | .14  .056 | -.07  .360 | .11  .153 | -.04  .548 |
| MLQ-P  change 15 | r  Sig. | **.54^**^**  **<.001** | -.32^**^  <.001 | **.54^**^**  **<.001** | .42^**^  <.001 | .25^**^  <.001 | 1 | -.37^**^  <.001 | .31^**^  <.001 | .39^**^  <.001 | .31^**^  <.001 | .30^**^  <.001 | .22^**^  .002 | .22^**^  .002 | .13  .071 |
| AAQ-II change 15 | r  Sig. | **-.54^**^**  **<.001** | .34^**^  <.001 | **-.46^**^**  **<.001** | -.35^**^  <.001 | -.37^**^  <.001 | -.37^**^  <.001 | 1 | -.41^**^  <.001 | **-.47^**^**  **<.001** | **-.43^**^**  **<.001** | -.27^**^  <.001 | -.18^*^  .013 | -.19^*^  .011 | -.12  .108 |
| BRS  change 15 | r  Sig. | **.36^**^**  **<.001** | -.13  .075 | .28^**^  <.001 | .16^*^  .031 | .22^**^  .003 | .31^**^  <.001 | -.41^**^  <.001 | 1 | .29^**^  <.001 | .23^**^  .001 | .25^**^  <.001 | .19^**^  .010 | .06  .388 | .07  .349 |
| CAMS-R change 15 | r  Sig. | **.48^**^**  **<.001** | -.30^**^  <.001 | .40^**^  <.001 | .25^**^  .001 | .20^**^  .007 | .39^**^  <.001 | **-.47^**^**  **<.001** | .29^**^  <.001 | 1 | .40^**^  <.001 | .32^**^  <.001 | .15*  .046 | .08  .279 | .22^**^  .003 |
| SCS  change 15 | r  Sig. | **.47^**^**  **<.001** | -.32^**^  <.001 | .40^**^  <.001 | .32^**^  <.001 | .29^**^  <.001 | .31^**^  <.001 | **-.43^**^**  **<.001** | .23^**^  .001 | .40^**^  <.001 | 1 | .34^**^  <.001 | .06  .431 | .11  .125 | .16^*^  .035 |
| GQ-6  change 15 | r  Sig. | **.34^**^**  **<.001** | -.15^*^  .044 | .28^**^  <.001 | .16^*^  .029 | .14  .056 | .30^**^  <.001 | -.27^**^  <.001 | .25^**^  <.001 | .32^**^  <.001 | .34^**^  <.001 | 1 | .19^*^  .009 | .16^*^  .031 | .17^*^  .018 |
| STS-U  change 15 | r  Sig. | **.21^**^**  **.003** | .03  .661 | .16^*^  .032 | .10  .195 | -.07  .360 | .22^**^  .002 | -.18^*^  .013 | .19^**^  .010 | .15*  .046 | .06  ,431 | .19^*^  .009 | 1 | .25^**^  .001 | .19^**^  .009 |
| SpREUK-SF-T change15 | r  Sig. | **.21^**^**  **.005** | -.08  .268 | .25^**^  .001 | .17^*^  .023 | .11  .153 | .22^**^  .002 | -.19^*^  .011 | .06  .388 | .08  .279 | .11  .125 | .16^*^  .031 | .25^**^  .001 | 1 | .13  .088 |
| SCBCS  change 15 | r  Sig. | .06  .442 | .09  .222 | .13  .070 | .14  .059 | -.04  .548 | .13  .071 | -.12  .108 | .07  .349 | .22^**^  .003 | .16^*^  .035 | .17^*^  .018 | .19^**^  .009 | .13  .088 | 1 |
| *Note*. Each cell contains the Pearson correlation coefficient (r) and the p-value. N = 185. Cases are excluded pairwise. Correlations with WEMWBS are highlighted in dark grey and non-significant correlations are provided in grey. Medium to large sized correlations with WEMWBS as well as the strongest 5 pairwise correlations are shown in **bold**. No corrections are done for multiple comparisons as these analyses were done for explorative purposes.  *. Correlation is significant at the .05 significance level (2-tailed) \| **. Correlation is significant at the .01 significance level (2-tailed) | | | | | | | | | | | | | | | |
