# Supplementary material for: Sustained, Multifaceted Improvements in Mental Well-Being Following Psychedelic Experiences in a Prospective Opportunity Sample
Source: Front Psychiatry. 2021 Jun 29;12:647909. doi: 10.3389/fpsyt.2021.647909 (PMC8277190; doi:10.3389/fpsyt.2021.647909)
Supplement: Supplementary file 5 [file Table_5.docx]

| Supplementary Material: Table 5.  *Correlation matrix of main measures at baseline (TP1), Bonferroni corrected for multiple comparisons.* | | | | | | | | | | | | | | | |
| --- | --- | --- | --- | --- | --- | --- | --- | --- | --- | --- | --- | --- | --- | --- | --- |
|  | | WEMWBS | QIDS-SR_16_ | RSE | LOT-R | TIPI-ES | MLQ-P | AAQ-II | BRS | CAMS-R | SCS | GQ-6 | STS-U | SpREUK-SF-T | SCBCS |
| WEMWBS | *r*  *Sig.* | 1 | **-.64^**^**  **<.001** | **.76^**^**  **<.001** | **.64^**^**  **<.001** | **.57^**^**  **<.001** | **.55^**^**  **<.001** | **-.65^**^**  **<.001** | **.58^**^**  **<.001** | **.62^**^**  **<.001** | **.59^**^**  **<.001** | **.56^**^**  **<.001** | .26^**^  <.001 | .23^**^  <.001 | .20^**^  <.001 |
| QIDS-SR_16_ | *r*  *Sig.* | **-.64^**^**  **<.001** | 1 | -.65^**^  <.001 | -.54^**^  <.001 | -.57^**^  <.001 | -.35^**^  <.001 | .62^**^  <.001 | -.49^**^  <.001 | -.48^**^  <.001 | -.51^**^  <.001 | -.39^**^  <.001 | -.07  1.000 | -.08  1.000 | .02  1.000 |
| RSE | *r*  *Sig.* | **.76^**^**  **<.001** | -.65^**^  <.001 | 1 | **.70^**^**  **<.001** | .62^**^  <.001 | .52^**^  <.001 | **-.69^**^**  **<.001** | .53^**^  <.001 | .61^**^  <.001 | .57^**^  <.001 | .49^**^  <.001 | .22^**^  <.001 | .20^**^  <.001 | .15^*^  .017 |
| LOT-R | *r*  *Sig.* | **.64^**^**  **<.001** | -.54^**^  <.001 | **.70^**^**  **<.001** | 1 | .56^**^  <.001 | .42^**^  <.001 | -.61^**^  <.001 | .53^**^  <.001 | .50^**^  <.001 | .46^**^  <.001 | .50^**^  <.001 | .23^**^  <.001 | .22^**^  <.001 | .14^*^  .026 |
| TIPI-ES | *r*  *Sig.* | **.57^**^**  **<.001** | -.57^**^  <.001 | .62^**^  <.001 | .56^**^  <.001 | 1 | .31^**^  <.001 | **-.67^**^**  **<.001** | .56^**^  <.001 | .54^**^  <.001 | .43^**^  <.001 | .34^**^  <.001 | .10  .804 | .08  1.000 | .06  1.000 |
| MLQ-P | *r*  *Sig.* | **.55^**^**  **<.001** | -.35^**^  <.001 | .52^**^  <.001 | .42^**^  <.001 | .31^**^  <.001 | 1 | -.41^**^  <.001 | .32^**^  <.001 | .48^**^  <.001 | .41^**^  <.001 | .50^**^  <.001 | .39^**^  <.001 | .42^**^  <.001 | .28^**^  <.001 |
| AAQ-II | *r*  *Sig.* | **-.65^**^**  **<.001** | .62^**^  <.001 | **-.69^**^**  **<.001** | -.61^**^  <.001 | **-.67^**^**  **<.001** | -.41^**^  <.001 | 1 | **-.66^**^**  **<.001** | -.64^**^  <.001 | -.53^**^  <.001 | -.45^**^  <.001 | -.10  .972 | -.07  1.000 | -.07  1.000 |
| BRS | *r*  *Sig.* | **.58^**^**  **<.001** | -.49^**^  <.001 | .53^**^  <.001 | .53^**^  <.001 | .56^**^  <.001 | .32^**^  <.001 | **-.66^**^**  **<.001** | 1 | .53^**^  <.001 | .41^**^  <.001 | .37^**^  <.001 | .12  .250 | .08  1.000 | .11  .379 |
| CAMS-R | *r*  *Sig.* | **.62^**^**  **<.001** | -.48^**^  <.001 | .61^**^  <.001 | .50^**^  <.001 | .54^**^  <.001 | .48^**^  <.001 | -.64^**^  <.001 | .53^**^  <.001 | 1 | .43^**^  <.001 | .41^**^  <.001 | .23^**^  <.001 | .20^**^  <.001 | .16^*^  .003 |
| SCS | *r*  *Sig.* | **.59^**^**  **<.001** | -.51^**^  <.001 | .57^**^  <.001 | .46^**^  <.001 | .43^**^  <.001 | .41^**^  <.001 | -.53^**^  <.001 | .41^**^  <.001 | .43^**^  <.001 | 1 | .51^**^  <.001 | .15^*^  .007 | .12  .144 | .19^**^  <.001 |
| GQ-6 | *r*  *Sig.* | **.56^**^**  **<.001** | -.39^**^  <.001 | .49^**^  <.001 | .50^**^  <.001 | .34^**^  <.001 | .50^**^  <.001 | -.45^**^  <.001 | .37^**^  <.001 | .41^**^  <.001 | .51^**^  <.001 | 1 | .36^**^  <.001 | .30^**^  <.001 | .36^**^  <.001 |
| STS-U | *r*  *Sig.* | .26^**^  <.001 | -.07  1.000 | .22^**^  <.001 | .23^**^  <.001 | .10  .804 | .39^**^  <.001 | -.10  .972 | .12  .250 | .23^**^  <.001 | .15^*^  .007 | .36^**^  <.001 | 1 | **.81^**^**  **<.001** | .43^**^  <.001 |
| SpREUK-SF-T | *r*  *Sig.* | .23^**^  <.001 | -.08  1.000 | .20^**^  <.001 | .22^**^  <.001 | .08  1.000 | .42^**^  <.001 | -.07  1.000 | .08  1.000 | .20^**^  <.001 | .12  .144 | .30^**^  <.001 | **.81^**^**  **<.001** | 1 | .33^**^  <.001 |
| SCBCS | *r*  *Sig.* | .20^**^  <.001 | .02  1.000 | .15^*^  .017 | .14^*^  .026 | .06  1.000 | .28^**^  <.001 | -.07  1.000 | .11  .379 | .16^**^  <.001 | .19^**^  <.001 | .36^**^  <.001 | .43^**^  <.001 | .33^**^  <.001 | 1 |
| *Note*. Each cell contains the Pearson correlation coefficient (r), followed by the Bonferroni-adjusted p-value - corrected for multiple comparisons.  *N* = 654. Cases are excluded pairwise. Correlations with WEMWBS (general well-being) are highlighted in dark grey. Non-significant correlations are provided in grey. Medium to large sized correlations with WEMWBS as well as the strongest 5 pairwise correlations are shown in **bold**.  *. Correlation is significant at the .05 significance level (2-tailed) **. Correlation is significant at the .01 significance level (2-tailed) | | | | | | | | | | | | | | | |
