# Supplementary figures and images for: Sustained, Multifaceted Improvements in Mental Well-Being Following Psychedelic Experiences in a Prospective Opportunity Sample
Source: Front Psychiatry. 2021 Jun 29;12:647909. doi: 10.3389/fpsyt.2021.647909 (PMC8277190; doi:10.3389/fpsyt.2021.647909)

A.

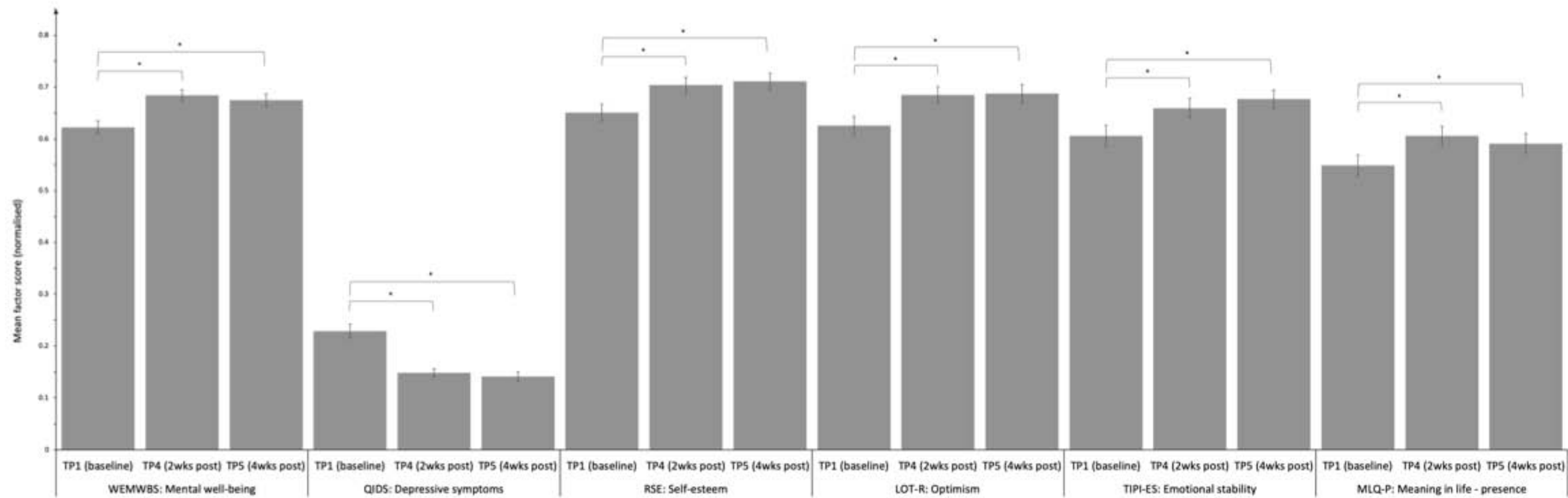

B.

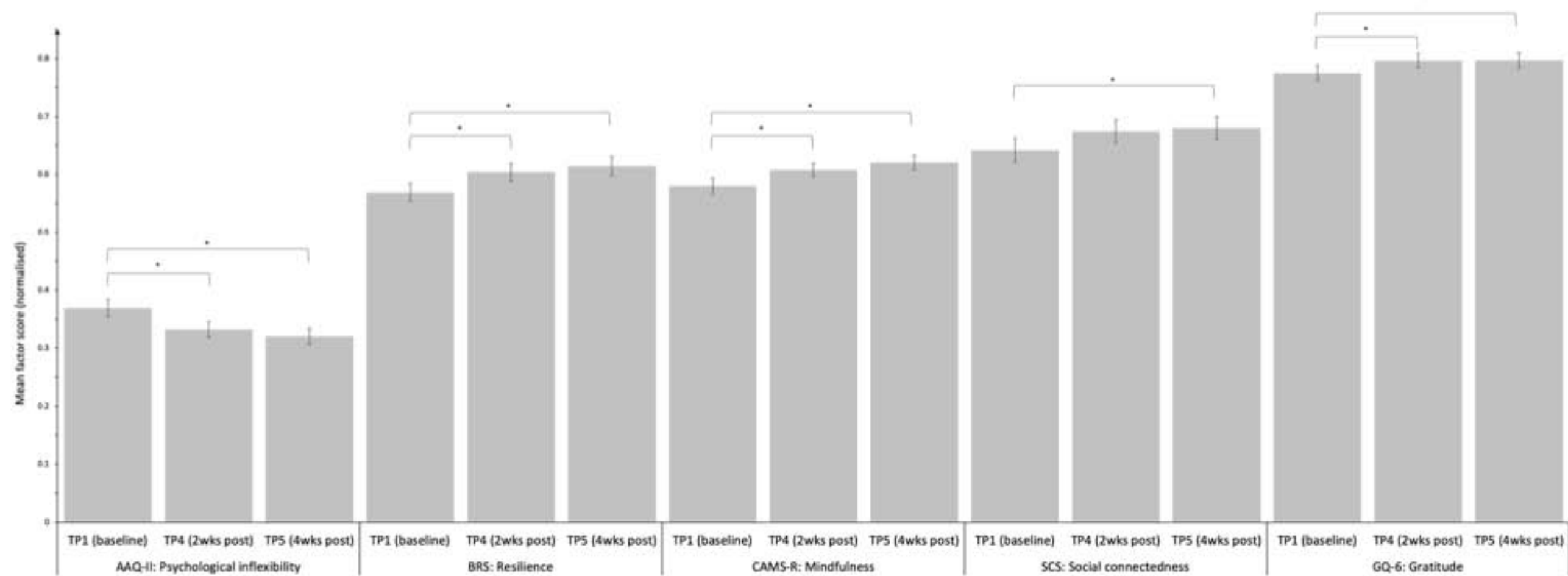

C.

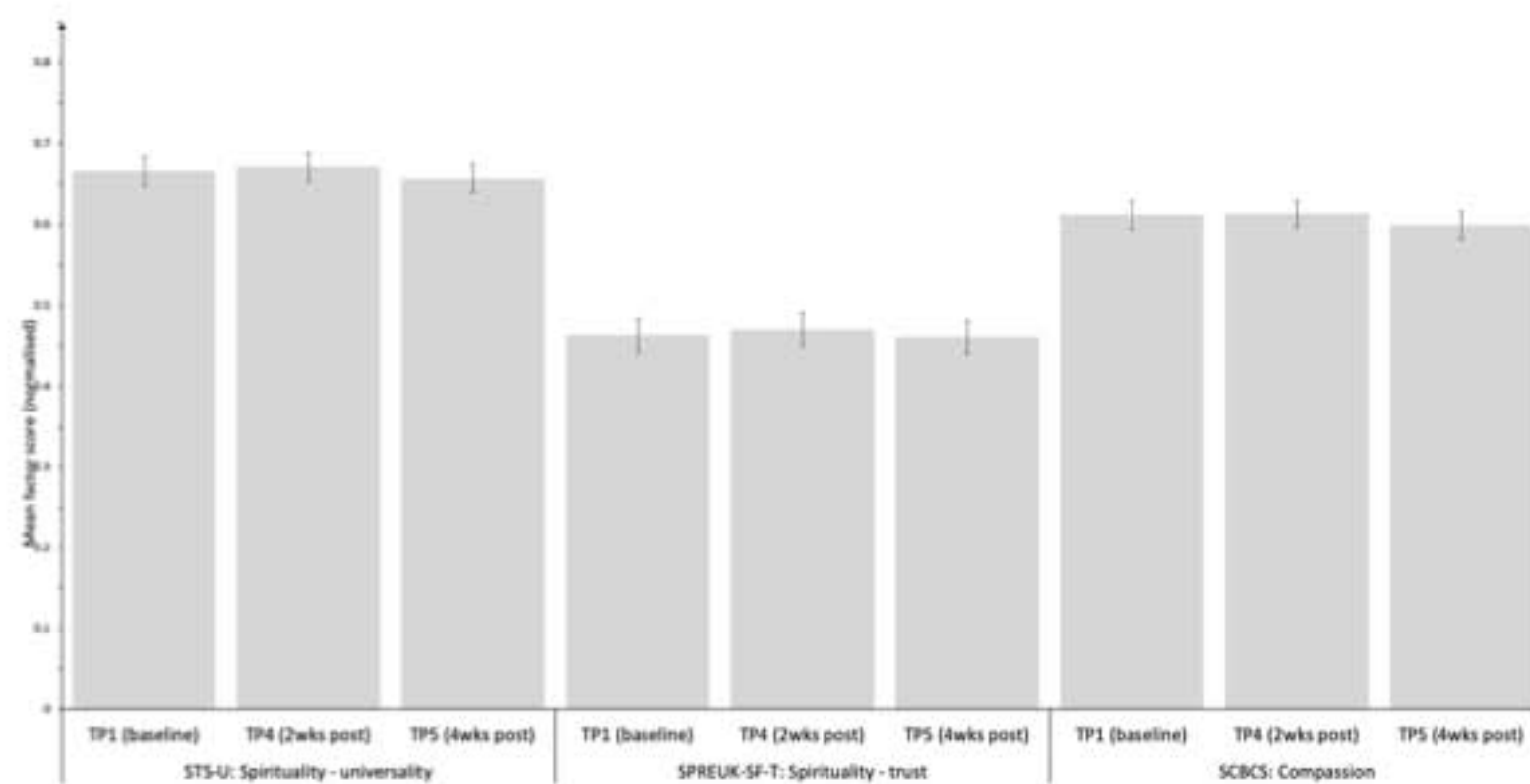

Supplement: Supplementary file 6 [file Image_1.pdf]
